# The ICU Bridge Program: volunteers bridging medicine and people together

**DOI:** 10.1186/s13054-022-04209-4

**Published:** 2022-11-08

**Authors:** Sarah Petrecca, Adrian Goin, David Hornstein, Milanka Stevanovic, Adamo Anthony Donovan

**Affiliations:** 1 Faculty of Medicine and Health Sciences, McGill University, 3655 Promenade Sir-William-Osler, Montreal, Canada; 2 Faculty of Engineering, McGill University, Montreal, Canada; 3 Department of Ophthalmology, Albany Medical Center, Albany, NY USA

**Keywords:** Intensive care unit, Volunteer, Visitation, Whole person care

## Abstract

**Background:**

The intensive care unit (ICU) is an emotionally taxing environment. Patients and family members are at an increased risk of long-term physical and psychological consequences of critical illness, known collectively as post-intensive care syndrome (PICS). These environmental strains can lead to a high incidence of staff turnover and burnout.

**Aim:**

The ICU Bridge Program (ICUBP) is a student-led organization that attempts to mitigate these stressors on patients, family, and staff, by assigning university volunteers to ICUs across Montreal.

**Setting:**

ICU.

**Participants:**

ICU volunteers, staff, patients, and families.

**Program description:**

The ICUBP volunteers support staff by orienting patients and families, while using effective communication strategies to provide comfort and promote a calm environment. The presence of volunteer visitors is helpful to patients who do not have the support of family members and/or friends. The program provides students with profound learning experiences by allowing them to shadow multidisciplinary teams, gaining a privileged and varied exposure to an acute medical environment, while developing their communications skills.

**Program evaluation:**

The program reassesses its methods and impact via internal student-designed surveys distributed on a yearly basis to staff and volunteers.

**Discussion:**

Research is warranted to assess the impact of the program on ICU patients, visitors, staff, and volunteers.

## Introduction

The intensive care unit (ICU) is an emotionally taxing environment. People from all walks of life are thrown into a distressing and tumultuous situation with very little control or guidance. For both patients and family members, the events that occur in the ICU can have many negative long-term psychologic and physical effects [1].

Patients in the ICU are often sedated and receive mechanical ventilation, making it difficult for them to recall the events leading up to their hospital admission, as well as their treatment and recovery process. Up to 80% of patients will experience hallucinations and delusional memories of their ICU stay [2]. As a result, many develop post-intensive care syndrome (PICS), with symptoms of post-traumatic stress such as excessive anxiety, flashbacks, emotional lability, frequent triggers, and depression [3]. For patients, mental and physical stress can worsen their medical condition, extend the length of their treatment, and ultimately endanger their lives [1]. One effective solution to this problem is visitation. Visitation allows patients to stay in touch with their family members and friends, to be aware of events happening outside of the hospital, and it has been shown to have a positive effect on the patient's condition [4].

As a result of the intimidating and complex nature of the ICU, its restricted access, and the limited ability to interact with patients, family members experience difficult emotions alongside their ill loved one. In fact, as many as 33% of family members can develop long-term psychologic consequences, known collectively as PICS-Family [3]. There is evidence that healthy visitors show an increased likelihood of developing depression and of having negative perceptions of the ICU following the discharge of a family member [5]. Given the importance of patient and family-centered care, it is of vital importance to recognize and address this issue during the ICU experience to ensure that family members receive deserved attention when illness strikes their loved ones.

Many issues arise when attempting to address these problems. Hospital staff recognize the importance of compassionate care and do their best to support the mental health of their patients and family members. However, patient care is taxing, and the added demand to attend to the social needs of patients and their families may contribute to staff burnout [6]. It is for these reasons that facilitating the important role of visitation, while at the same time minimizing any added burden on health care workers is an incredibly important task.

The introduction of volunteers into healthcare contexts can address these issues and studies (Table 1) have demonstrated that volunteer interventions are an efficient method to:

1. Increase patient and team morale by dedicating time to one-on-one patient interactions, increasing socialization, reducing anxiety, and alleviating staff workload.
2. Improve quality of care and reduce adverse health outcomes, such as length of stay, falls, and improve food intake and mobilization.

**Table 1:** Literature review of volunteers in healthcare

Department	Publication	Description	Outcomes
Neonatal intensive care unit (NICU)	Thompson et al. [17]	Comfort Club is an initiative in which student volunteers provide positive touch and support for infants in the NICU	Provides: 1. Considerable learning opportunities for volunteers 2. Support to parents and staff
Palliative Care	Bloomer et al. [18]	Study aimed to understand the role and experience of palliative care volunteers	Volunteers were appreciated for providing psychosocial support and were found to be a good addition to the healthcare team
Mental Health	Shipley et al. [19]	Assessed the effects of a companion program on: 20 college student volunteers 21 mental patients	Companionship was beneficial to some patients. Hospital staff, student companions, and patients had positive feelings about the program and wanted it to continue
	Bateman et al. [20]	Addressed challenges faced by staff when providing care for patients with dementia and/or delirium. Volunteers were trained to support patients	The program was deemed feasible and an inexpensive way to improve the quality of care and significantly reduce length of stay for those with dementia and/or delirium
	Ervin et al. [21]	Addressed the opinions of nursing staff caring for patients with dementia/delirium following the introduction of a volunteer program	Improved patient care and time management for nursing tasks
	Shee et al. [22]	Evaluated the feasibility and staff, volunteer, and patient/caretaker acceptance of a volunteer program for patients with cognitive impairment undergoing inpatient rehabilitation	Patients, their caretakers, staff, and volunteers were satisfied with the program and believed it improved quality of care
	Sandhaus et al. [23]	Volunteer intervention aiming to prevent and reduce delirium in admitted patients 70 years or older	Nurses, patients, and their families were satisfied with the program. The use of volunteers is a cost-effective way to enhance the care of delirious elders in an inpatient setting
Pediatric Emergency	Steadman et al. [24]	Assessed the impact of university student volunteers on pediatric emergency room research outcomes	University student volunteers can facilitate research enrollment and recruit eligible patients into prospective clinical research studies
Adult In-Patient	Saunders et al. [25]	Instilled a volunteer program to minimize the potential adverse health outcomes of an aging acute inpatient population	Volunteer care and support with eating, drinking, mobilizing, and therapeutic activities can positively impact patient outcomes
	Green et al. [26]	Introduced volunteers to assess their impact on the prevalent issue of malnutrition in adult patients or resident populations in institutional care	Volunteers can improve mealtime care of adult patients or residents in institutional settings
	Walton et al. [27]	Malnutrition is prevalent in elderly hospitalized patients and is associated with adverse health outcomes. Assessed volunteer’s involvement in feeding to assist healthcare workers who lack time to ensure nutritional care of all patients	Patients who were assisted by volunteers had an increased protein and energy intake. Relative to nurses, volunteers socialized more with patients and encouraged them to eat more often
	Donoghue et al. [28]	Introduced volunteer companion-observers to prevent falls on a high-risk acute aged care ward	Fall rates decreased by 44% and no patients fell when volunteers were present. The presence of volunteers was appreciated by relatives of patients
	Baczynska et al. [29]	Immobility in hospitals is associated with poor healthcare outcomes in older patients, but mobility is often compromised due to staff time constraints. Investigated the effect of volunteers in helping with inpatient mobilization	The healthcare team was satisfied with this initiative and volunteers can be an asset

It is of critical importance to ensure that volunteers are adequately trained. Not only does this ensure patient safety and satisfaction, but it allows the volunteers to properly integrate into the healthcare team and feel confident in their position. Moreover, volunteers need to be provided with the tools necessary to safely navigate emotionally charged environments. Studies have shown that nursing turnover is linked to new ICU nurses experiencing high levels of anxiety associated with lack of hospital knowledge and experience and the gap between their educational and practical work [7, 8]. Similarly, other members of the ICU staff, such as physical therapists and residents, have felt underprepared going into the ICU environment [9]. To mitigate this, studies have shown that high-ranking competencies such as socialization, communication with colleagues and supervisors, as well as active listening to patients led to early success in the medical workforce [7, 10]. Another potential solution is to give prospective staff more exposure and experience inside the hospital through simulations and shadowing programs, as this is not always included in the standard curricula due to time limitations [9, 11, 12].

The ICU Bridge Program (ICUBP) (www.icubridgeprogram.org) was operationalized in 2016 by McGill students MS and AAD based on an idea and concept from, and under the faculty guidance of DH. This program assigns university students to hospital ICUs in the Montreal area to help families navigate the hospital environment and support their journey through their family member’s critical illness. The volunteers assist ICU staff by regulating the flow of visitors in the ward and being emotionally supportive to their needs. They may also communicate simple information to visitors, such as updates on care, schedules, and patient movement to and from the ICU.

This program differs from previously described critical care volunteer programs [13] and other volunteering programs (Table 1) in that it is organized primarily by university students, its volunteer base is open to all undergraduate students regardless of faculty, and it offers combined volunteering and shadowing opportunities. As such, this program aims to further alleviate staff workload by supporting the emotional wellbeing of patients and family members, while providing a rich educational experience for its volunteers. Its student-led executive team removes nearly all responsibilities that a volunteering program of such caliber would impose on hospital medical and administrative/volunteer staff.

## Setting and participants

**Fig. 1:**
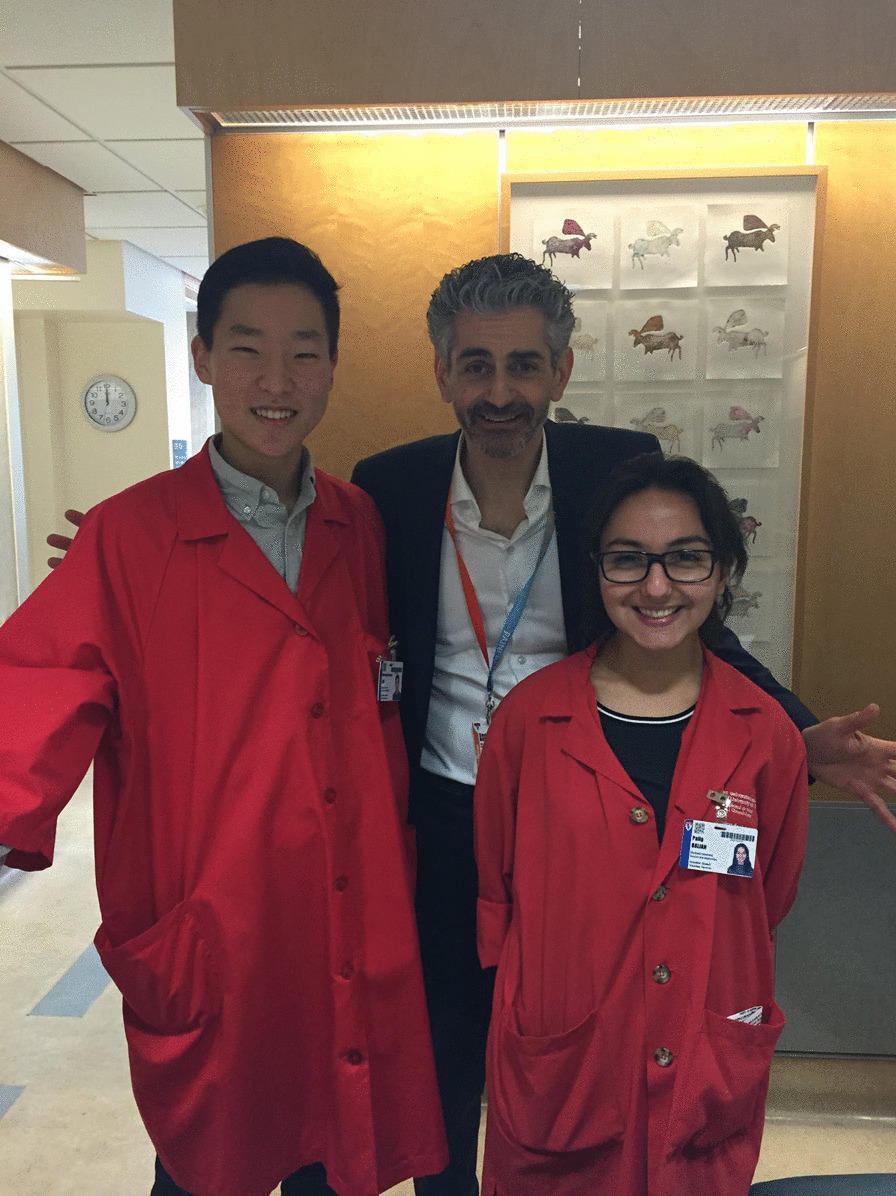
The Intensive Care Unit Bridge Program’s 1st volunteers with co-founder Dr. David Hornstein (March, 2016)

**Fig. 2:**
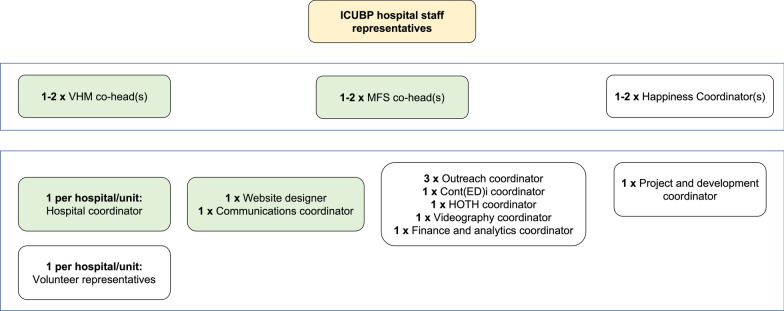
Structure of the Intensive Care Unit Bridge Program’s executive team. ICUBP, Intensive Care Unit Bridge Program; VHM, Volunteer and Hospital Management; MFS, Marketing, Fundraising, and Socials; Cont(ED)I, continuing education and equity, diversity, and inclusion; HOTH, humans of the hospital. *Box colours*: Yellow = Hospital staff representatives and allies of the ICUBP, Green = Minimal viable executive team structure, White = Ideal executive team structure (in addition to Green). Descriptions: *ICUBP hospital staff representatives:* These are members of the volunteer office and ICU staff (nurses, social workers, doctors, etc.) that help the ICUBP executives integrate the program into their hospital/unit and inform their colleagues about the program. They are the go-to people for ICU staff, volunteers, and executives for matters regarding the ICUBP. *Co-head(s):* Each team has co-head(s) that coordinate their activities with their fellow co-head(s) while managing their team’s efforts. Co-head(s) simultaneously hold positions within their team (i.e., a co-head may also be a hospital coordinator for the VHM team or a communications coordinator for the MFS team). *VHM team:* Coordinates everything with regard to the volunteer workflow (recruitment, applications, interviews, orientations, scheduling, and end of semester transitions) and collaborates with hospital volunteer offices and ICU staff. (1) *Hospital coordinator:* Responsible for volunteer administrative tasks, workflows, and timelines. (2) *Volunteer representative:* Responsible for the volunteers’ experiences within the ICU via in-person and remote check-ins. During these volunteer check-ins, they will also communicate with the ICUBP hospital staff representatives. (1) The coordinator and representative roles largely overlap, but the coordinator is generally the more experienced executive and understands more of the ICUBP logistics and timelines. *MFS team:* Responsible for a variety of tasks related to increasing volunteer recruitment, informing volunteers, staff, the public regarding the ICUBP’s activities, and raising funds to ensure the program is self-sufficient. (1) *Website designer:* Responsible for updating the program’s website (www.icubridgeprogram.org), which is the volunteers’ and community’s first impression of the program and crucial for the volunteer application process. (2) *Communications coordinator:* Responsible for the program’s email correspondence and social media. (3) *Outreach coordinator:* Responsible for reaching out to universities and the community to help recruit volunteers and raise funds to support the program (bake sales, attending university activity nights, class announcements, etc.). (4) *Cont(ED)i coordinator:* Responsible for assessing and promoting the diversity of the ICUBP’s volunteers and executive team through our equity survey (Fig. 3), as well as coordinating workshops with experts and invited guests for our volunteers regarding the intensive care unit or matters related to equity. (5) *HOTH coordinator:* Responsible for creating our photoblog (https://www.icubridgeprogram.org/humans-of-the-hospital, inspired by https://www.humansofnewyork.com/) by interviewing and photographing our ICUBP stakeholders in order to bring to light insightful stories and experiences from our ICU staff, patients, visitors, volunteers, and executives. (6) *Videography coordinator:* Responsible for filming, editing, and creating promotional and informational videos regarding the program. (7) *Finance and analytics coordinator:* Responsible for record keeping of the program’s financials and the creation and analysis of our program surveys. *Happiness team:* Happiness coordinators are co-heads who are responsible for: on-boarding new executives through orientations, increasing collaboration and communication between the different teams and their members, and checking-in on the interpersonal and emotional well-being of the ICUBP executive. They help organize executive and volunteer social gatherings and team/community bonding exercises along with the outreach coordinators. (1) *Project and development coordinator:* Responsible for projects like the ICU journals and for the long-term sustainability and efficiency of the ICUBP by updating the program’s standard operating procedure manual

The ICUBP started at the Montreal General Hospital in 2016 (Fig. 1) and is currently operational in 4 different intensive care units (3 adult and 1 pediatric) in the Montreal area. The ICUBP student administration coordinates over 160 volunteers. The program is entirely run by an executive team of 15 + university students (Fig. 2). The ICUBP is self-funded through student initiatives coordinated by a dedicated branch of the executive team. On site offerings to family members such as refreshments and incidental small gifts are funded through a separate fund, The Lauren Alexander Family Support fund.

**Fig. 3:**
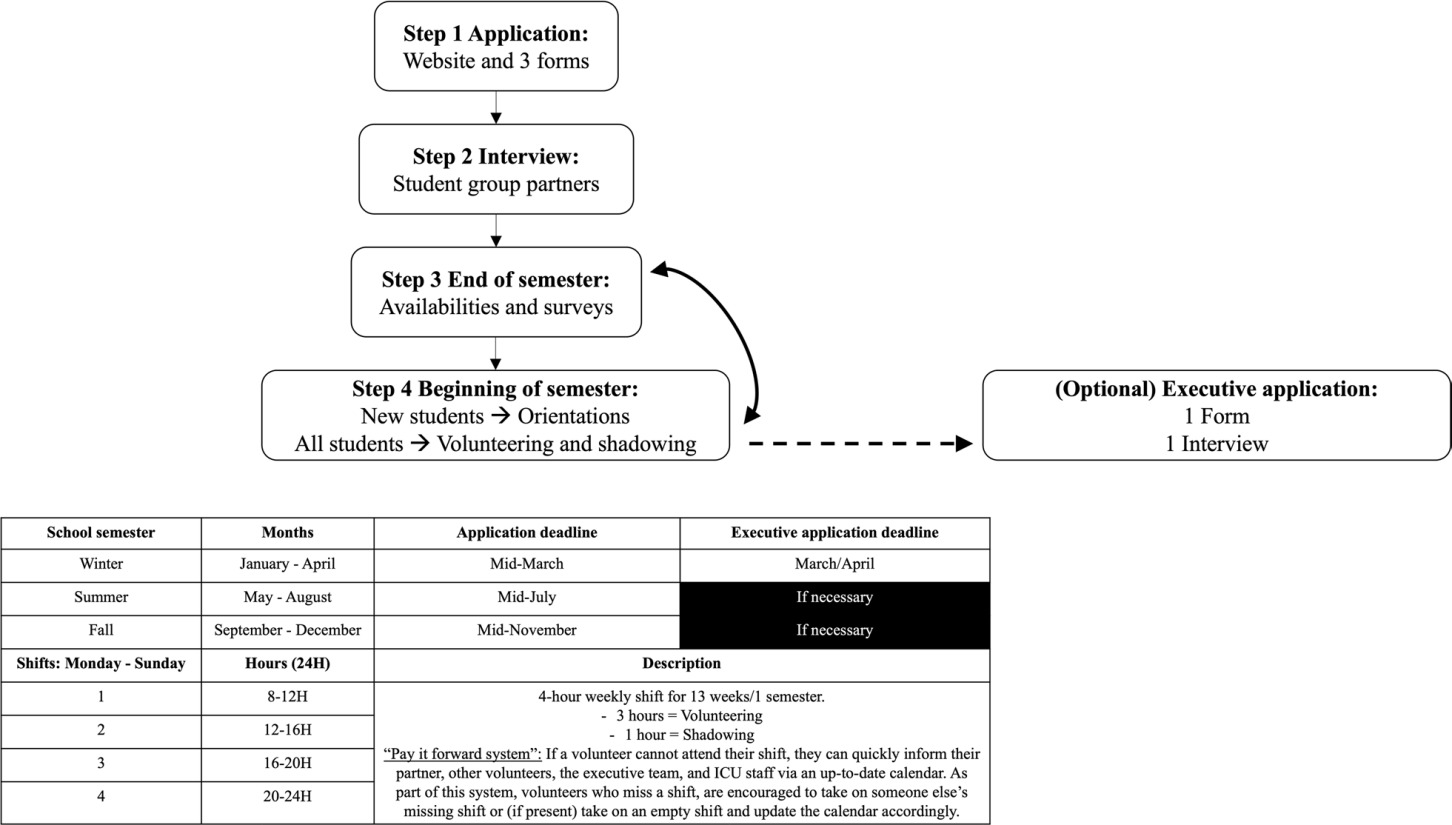
Volunteer and executive workflow and protocol. Description: Steps: (1) The ICUBP applicant reviews the ICUBP’s website (https://www.icubridgeprogram.org/) to ensure they fulfill the program’s requirements and have their documentation and required information in order. The applicant fills out 2 separate forms (~ 20 min) that automatically generate and pre-populate the required PDF documents for the ICUBP and each of its hospital, minimizing the program’s focus on form completion. The 3rd form is optional and is a 12-min anonymous equity and diversity survey to assess the composition of students applying to the program. (2) If the application is complete and all the appropriate information is given, the application is approved by the ICUBP team and the program’s university student group partners interview the applicant according to a standardized form developed by the ICUBP. Once the interview is completed and the form submitted, the ICUBP executive team makes the final decision on all applicants. For additional details on the automations and integrations involved in Steps 1 and 2, please refer to: https://doi.org/10.36834/cmej.73818. Steps 3 and 4 are the ICUBP’s transitional period to accommodate the changing availabilities and schedules of students that occur between semesters. (3) If the applicant is accepted into the program, they and the current cohort of volunteers will be invited to fill out our semester survey and provide their availabilities for the upcoming semester on a rolling admissions basis due to their being limited spots/shifts available within the program and hospitals. Volunteer priority goes as follows: current volunteers > returning volunteers > new volunteers. The application deadline for new volunteers is the 20th of the 3rd month of every semester in order to provide some time for applicants to be reviewed, interviewed, and give their availabilities. Though this varies according to each unit/hospital’s needs, the ICUBP offers 28 possible 4-h weekly shifts, which further increases the accessibility of the program as it can accommodate a variety of schedules. Students are paired up primarily according to their availabilities, but if the availabilities allow for it, the ICUBP executive team does their best to pair new volunteers with more experienced ones and considers other factors to optimize volunteer experience and program impact. (4) New volunteers or students that wish to switch to a new site must attend orientations that are hosted by the ICUBP Hospital Coordinator and Volunteer Representative, with the help of the ICUBP’s hospital staff representatives, which include the hospital’s volunteer manager who completes the necessary in-person volunteer registration tasks (police checks, hospital IDs, etc.) and ICU staff who help with touring the ICU. Volunteers can begin volunteering and shadowing. (5) (Optional) If the volunteer wishes, they can apply to the ICUBP executive team when the application form opens at the end of the Winter semester, though there may be exceptional recruitment periods throughout the year depending on the executive team’s needs. Generally, the ICUBP recruits and gives priority to applicants that have volunteered previously with the ICUBP, particularly for VHM positions, where familiarity with volunteering and the ICU environment is crucial. However, executive positions are open to the general university population. Executive applicants must fill out 1 form and if approved, go on to be interviewed by the executive team, who are guided by an interview form that standardizes the executive application system according to the applicant’s position of interest

Volunteers (Fig. 3) work in dyads to ensure that constant support is provided to patients, their families, the staff, and each other. They participate in a weekly 4-h shift for a minimum commitment of 13 weeks which aligns with university semesters (Winter, Summer, and Fall). This consistency creates a schedule that ICU staff and volunteer offices can easily follow and helps develop professional relationships between volunteers and staff, which further facilitates the positive learning environment the ICUBP fosters.

Indeed, the ICUBP not only aims to help patients, families, and staff, but also offers students the chance to learn more about the healthcare system and improve their communication skills. Through their weekly shifts and multiple interactions with patients, visitors, and staff, volunteers can better understand the inner workings of the hospital environment, and more specifically the ICU and its multidisciplinary nature. The student volunteers can improve their ability to speak with empathy, manage confidential information, and navigate difficult emotional situations. These are assets that will serve them in all their future endeavors, whether medical or otherwise.

A central and innovative facet of this program is the weekly combined volunteering and shadowing components that offers students the opportunity to follow and learn from health professionals during a shift. Shadowing is a much sought-after activity for interested students; however, its availability is exceptionally limited. As such, this structured volunteering-based program offers the opportunity for students to gain hospital experience by shadowing medical professionals of the ICU while at the same time fulfilling an important and meaningful volunteering role.

### Program description

Volunteers fulfill multiple roles within the ICU. They help visitors by welcoming them into the ward and orienting them toward the patient or staff they wish to see, thus increasing communication within the ICU. They help lend a non-medical and friendly ear to those in need and offer different amenities (water, snacks, plushies, coloring books, etc.) that are supplied by The Lauren Alexander Family Support fund to visitors and their children who often stay alongside their sick loved ones for extended periods of time. Their role in relaying information between staff and family members is crucial in improving the ICU’s environment, as it allows staff to concentrate on the quality of care they provide.

In some circumstances, family members can find it difficult to be present for their loved ones due to work constraints and other familial responsibilities. There are also patients who do not have visitors, because they lack contacts within the city/country, are elderly and may not have as many visitors as younger patients, or for other reasons. With visitation being considered an essential aspect of reducing delirium during patient care, volunteers can play a crucial role in such situations as they can reduce the emotional pressure felt by families and help patients and staff by keeping them company [14]. Depending on the patient’s condition, volunteers are available to engage in discussions or even play games, encouraging them to be more physically or mentally active.

### Program evaluation

The ICUBP was created and implemented by undergraduate university students, with the overarching objectives to provide volunteering and shadowing experience to students while providing important service to both patients, family members, and hospital staff. It was not designed as a research project and to date has not been implemented nor analyzed in an empirical fashion. However, observations since inception have led to an understanding that this area is rife for investigation. As a program grounded on patient and family centered care values, on family engagement, and on emotional wellbeing of family and staff, structured formal analyses of overall family/visitor staff satisfaction with the program should be conducted with validated measures. The effects the program has on perceived unit workload and stress levels among personnel, and finally its impact on the volunteers themselves are also areas of great interest for future research. Our team has recognized the importance of formal collaboration with expertise in the field to pursue these questions further, and such collaboration is sought through DH and the division of General Internal Medicine at McGill.

Evaluations of the program to date have been in the form of qualitative and quantitative anonymous student-designed surveys distributed internally to all volunteers and staff every 4 to 12 months. These surveys attempt to quantify program satisfaction and impact, and the information is used to assess the current systems in place and make improvements where needed. Data from surveys completed by volunteers and staff over a 6-year period have been used to reorganize shifts to facilitate patient support, improve orientations to better prepare volunteers, and adapt to different high stress situations, such as the COVID-19 pandemic. In addition, volunteers and executives can provide anonymous feedback anytime throughout the year through an online form and executives are encouraged to complete a yearly review of the program’s efforts and/or an exit report when they leave the team.

More recently, an equity and continuing education coordinator position has been added to the executive team. This member aims to highlight the importance of diversity in the program through the implementation of an equity survey. This survey evaluates volunteer demographics and the representation of groups that are underrepresented within the medical field, such as black, indigenous, low socioeconomic, and rural peoples. The form then requests feedback regarding the program’s accessibility for these groups.

A new project for the ICUBP, involvement with the hospital’s Intensive Care Journal program, has been implemented by DH to continue improving the program’s impact. The objective of the journals is to help patients piece together their ICU experiences and “fill in the gaps of lost time.” In this project, families, staff, and volunteers can input entries in a non-medical context, such as:

- wishes and reflections
- kind messages,
- pictures taken during family visits,
- progress,
- updates on life outside of the hospital.

These elements have been shown to aid in patient and family member recovery [15]. The ICUBP’s volunteers help manage and encourage journal entries by explaining how the journal application works, assisting staff and family members with making entries, and by contributing content to the journals themselves. Volunteers offer an important perspective to the patient’s stay, particularly in instances where patients do not receive visitors. Once patients are discharged from the ICU, they have the option to have their journals gifted to them to help in their post-treatment recovery. These journals have been shown to reduce the incidence of mental illness following treatment in the ICU as they help patients understand and better integrate their experiences with their illness [15].

## Discussion

Supporting patients and family members through communication and compassionate care is essential to the recovery process and the ICUBP targets this [8]. Volunteers are managed by fellow student volunteers and incorporated where they are needed most—at the ICU front desks, waiting rooms, and at the bedside. The ICUBP also addresses situations in which patients may not have visitors and alleviates some of the pressure staff feel to compensate for these patients. Volunteers therefore act as the bridge between families, staff, and patients, supporting both ends by representing the hospital staff (within the realms of their training) while supporting the non-medical needs of the patients and families.

Although there are many positive aspects to the program, it can be stressful for volunteers. They often encounter difficult situations of helping meet the needs of family members while also supporting healthcare workers. Communicating with distraught family members can also be difficult for volunteers. To address this concern, the ICUBP trains its volunteers to properly address issues that may arise through volunteering. The executive team is constantly working with the ICU staff to ensure volunteers have the right tools to communicate effectively with patients, families, and staff. Each hospital site is assigned two dedicated senior team members as Volunteer Representative and Hospital Coordinator (Fig. 2), who support the volunteers on the terrain by providing hospital and unit-specific guidance and expertise.

There have been limitations to date in the ability to formally quantify the program’s impact on patients, family members, and staff. Although the student-run surveys and testimonials have provided valuable information about the program, these surveys suffer from the standard issues of self-selection and of being unable to truly quantify improved mental health of patients, families, and staff [16]. Further information on the program’s strengths, weaknesses, opportunities, and threats (SWOT analysis) can be found in Table 2.

**Table 2:** SWOT analysis

Strengths	Weaknesses
1. Volunteer program that is run by and tailored to university students and their needs 2. Introduces volunteers into a novel setting with minimal workload added to hospital staff 3. Program is self-sufficient financially 4. Increases communication between hospital staff and visitors, reducing visitor and staff stress and workload 5. Increased visitation and stimulation by volunteers particularly for non-sedated patients without or minimal visitors 6. Increases accessibility of shadowing opportunities to a more diverse university population 7. Multiple levels of emotional support for volunteers: fellow volunteers and their shift partner, student executive team, and ICUBP hospital staff representatives	1. Though minimized, the program requires involvement and partnerships with hospital staff 2. Implementation and evaluation have been non-empirical to date 3. Limitations on the diversity of volunteers, as students of low socioeconomic status may not have the resources to volunteer

Opportunities	Threats
1. Transfer and implementation of novel humanizing initiatives and programs between ICUs a. For example: implementing the Montreal Children’s Hospital’s Glass Door Project (https://www.thechildren.com/news-and-events/latest-news/glass-door-project) to the adult ICU sites 2. Implementation of ICU journals and impacting patients and visitors outcomes through student volunteers 3. Implementation of the program’s model into other hospital departments	1. Lack of staff buy-in to the program, 2. Negative emotional impact on students 3. Workload imposed on ICUBP volunteer student executives may be overwhelming

The ICUBP has been bridging the gap between staff and families for the last 6 years. Emotional support is an important aspect for the health of patients, their families, and staff. Through their participation in this program, students contribute to the humanity of the ICU environment, gain key clinical experience, and become individuals with stronger empathy and communication skills. Through surveys and data collection, the student-run executive team strives to continuously improve its program so that it meets the needs of the dynamic hospital environment. With more research on volunteer, staff, patient, and family satisfaction, this program has future potential to further innovate hospital ICU environments.

## Data Availability

Not applicable.

## Abbreviations

ICU: Intensive care unit
ICUBP: ICU Bridge Program
PICS: Post-intensive care syndrome
HOTH: Humans of the hospital
MFS: Marketing, fundraising, and socials
VHM: Volunteer and hospital management
SWOT analysis: Strengths, weaknesses, opportunities, and threats
Cont(ED)i: Continuing education and equity, diversity, and inclusion
NICU: Neonatal intensive care unit
